# Development and Pilot Study of myfood24 West Africa—An Online Tool for Dietary Assessment in Nigeria

**DOI:** 10.3390/nu16203497

**Published:** 2024-10-15

**Authors:** Chinwe Adaugo Uzokwe, Chiaka Charles Nkwoala, Bassey E. Ebenso, Sarah Beer, Grace Williams, Gideon Onyedikachi Iheme, Chihurumnanya Gertrude Opara, Rasaki A. Sanusi, Henrietta Nkechi Ene-Obong, Janet E. Cade

**Affiliations:** 1 Nutritional Epidemiology Group, School of Food Science and Nutrition, University of Leeds, Leeds LS2 9JT, UK; j.e.cade@leeds.ac.uk; 2 Department of Human Nutrition and Dietetics, Michael Okpara University of Agriculture, Umudike P.M.B. 7267, Nigeria; nkwoala.chiaka@mouau.edu.ng (C.C.N.); iheme.gideon@mouau.edu.ng (G.O.I.); oparagertrude101@gmail.com (C.G.O.); 3 Leeds Institute of Health Sciences, School of Medicine, University of Leeds, Leeds LS2 9JT, UK; b.e.ebenso@leeds.ac.uk; 4 Dietary Assessment Ltd., Nexus, Discovery Way, University of Leeds, Leeds LS2 3AA, UK; s.l.beer@myfood24.org (S.B.); g.williams@myfood24.org (G.W.); 5 Department of Food Studies, Nutrition and Dietetics, Uppsala University, 753 10 Uppsala, Sweden; 6 Department of Human Nutrition and Dietetics, University of Ibadan, Ibadan 200001, Nigeria; sanusiadegoke2003@gmail.com; 7 Department of Human Nutrition and Dietetics, University of Calabar, Calabar 540281, Nigeria; nkeneobong@gmail.com

**Keywords:** food composition tables, energy intake, nutrition assessment, nutrition app, usability study, digital tool, diet monitoring

## Abstract

Background and objective: Tools to accurately and efficiently measure dietary intake in Nigeria are lacking. We aimed to develop and assess the usability of a new online dietary assessment tool for Nigeria—myfood24 West Africa. Methods: We developed the myfood24 West Africa database using data from existing food composition tables, packaged foods labels and research articles. The development followed seven steps: identified data sources, selected foods, processed/cleaned the data, calculated the nutrient content of recipes, created and allocated portion sizes, quality-checked the database and developed food accompaniments. To pilot the tool, we recruited 179 university staff in Nigeria using a cross-sectional design. Usability was assessed using a questionnaire that included the System Usability Scale (SUS) and a feedback session. Results: The database included 924 foods, with up to 54 nutrients and 35 portion-size images allocated to foods. Sixty percent of the data were sourced from the 2019 West Africa Food Composition Table, 17% from back-of-pack labels of packaged foods, 14% from the 2017 Nigerian Food Composition Table, 5% from generated recipes and 4% from the published literature. Of the participants, 30% (n = 53) self-recorded their food intake, with a total of 1345 food and drink entries from both self- and interviewer-collected data. The mean SUS score of 74 (95% CI: 68,79) indicated good usability. The feedback showed that the tool was user-friendly, educational and included a variety of local foods. Conclusions: This new tool will enhance the dietary assessment of the Nigerian population. More work will expand coverage to include more foods from the region.

## 1. Introduction

An accurate and efficient assessment of a population’s diet is needed to understand the links between diet and health- and nutrition-related outcomes in population-based studies. The accuracy of the dietary assessment depends on the dietary assessment methods and tools used which are prone to measurement errors if not carefully and objectively selected [1]. Underlying food composition tables play an important role in estimating nutrient intake; however, they often do not include the range of foods available in a specific region, including packaged foods and culturally significant mixed dishes, especially in African countries [2,3], making it challenging to accurately monitor the impact of the nutrition transition on health. Furthermore, the limitation contributes to the existence of dietary data gaps and slows down progress in addressing nutrition problems [4,5,6,7]. Therefore, to accurately measure energy and nutrient intake, food composition databases need to be comprehensive to capture the full range of foods consumed in a specific population [8].

Dietary intake data in Africa are most commonly collected using 24 h recalls and food frequency questionnaires (FFQs) [9,10]. Traditionally the 24 h recalls are paper-based, interviewer-administered, time-consuming and expensive, and this limits their use in large national surveys in Nigeria [11]. Conversely, FFQs have remained the method of choice despite their limitations, but they may not give valid estimates of actual nutrient intakes [12]. Recently, technology-based 24 h recall tools have emerged and are reported to be reliable alternatives to traditional recording methods [13,14,15]. Nigeria is experiencing a large growth in the use of technology, with 55% having access to the internet in 2021, up from 1% in 2001 [16]. However, the increasing use of dietary analysis software lacks inclusion of portion sizes and common Nigerian foods [9]. This presents an opportunity to enhance dietary assessment methods in Nigeria.

myfood24 is an online dietary assessment with over 200,000 foods [17]; initially developed to support dietary research in the UK, myfood24 now includes international food composition databases to support dietary assessment of various populations around the world. While the usability of myfood24 was tested and found to be suitable for both British and non-British populations [18,19], its applicability in Nigeria has not been tested. Our study aimed to adapt myfood24 for West Africa, with a focus on Nigeria, and test its usability among Nigerian adults.

## 2. Materials and Methods

### 2.1. Developing myfood24 West Africa Database

**Figure 1:**
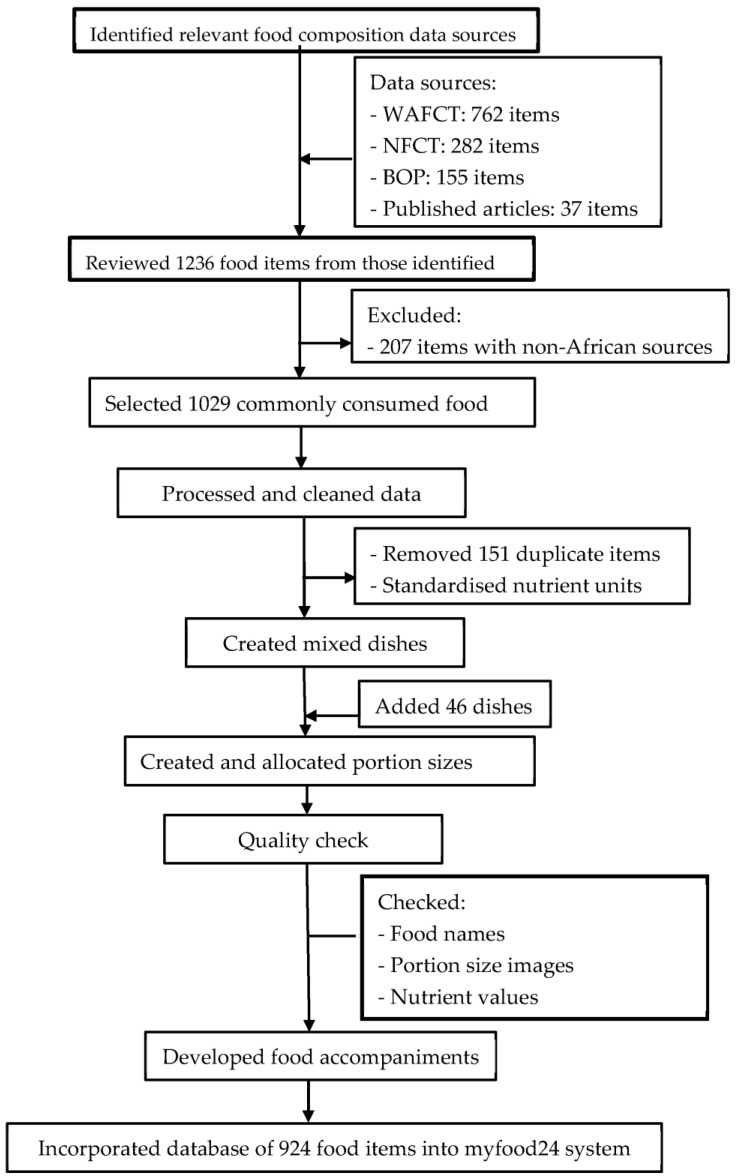
Flow chart of myfood24 West Africa development. WAFCT, West Africa Food Composition Table; NFCT, Nigerian Food Composition Table; BOP, back-of-pack labels of packaged foods.

Following guidelines [20], the steps taken to develop myfood24 West Africa are summarised below and in Figure 1.

#### 2.1.1. Identification of Relevant Food Composition Tables/Database

We used the following four (4) sources to compile the myfood24 West Africa database:

1. The 2019 West Africa Food Composition Table (WAFCT): This contains 1028 food items commonly consumed in West African countries including Nigeria with their corresponding 56 components, including 44 nutrients [21].
2. The 2017 Nigerian Food Composition Table (NFCT): This contains composition data for 282 foods consumed in Nigeria; data were sourced from chemical analysis and other food composition tables and contain up to 30 nutrients [22].
3. Back-of-pack labels of packaged foods: We took pictures of back-of-pack labels of packaged foods from one major supermarket in Abia State, another major supermarket in Enugu State and a local food store in Abia State. All locations are in southeast Nigeria. We also searched for composition data from the manufacturers’ websites.
4. Research articles: We searched the literature for the composition data of mixed foods and generic foods not present in the identified food composition tables above.

#### 2.1.2. Identification and Selection of Foods

All foods (in raw and cooked forms) identified in our food composition data sources 2 and 3 (above) were selected for the database. In addition, foods were selected from data sources 1 and 4 based on food names, synonyms, descriptions and ingredients known to the local researchers through reports of national food and nutrition surveys and Demographic and Health surveys and publications of commonly consumed foods [23,24]. Since Nigeria was our focus, we selected 762 foods from the WAFCT based on three criteria: they were also available in the NFCT, their food composition data were sourced from Nigeria or they are commonly consumed in Nigeria. Out of the 762 foods, we excluded 207 food items in the WAFCT that were sourced from the UK food composition tables as these foods will already be in the myfood24 system.

#### 2.1.3. Data Processing and Cleaning

The food database was created using a myfood24 data extraction template on Microsoft Excel. In cases where duplicate entries were found (from sources like NFCT and WAFCT), we prioritised the data with the most comprehensive nutrient analysis. We kept similar foods that were cooked by different cooking methods, e.g., “plantain, ripe, boiled” and “plantain, ripe, fried in oil” were both retained in the database. Nutrient units were reformatted to match the myfood24 formats, e.g., vitamin D was converted from IU to μg. When back-of pack labels provided nutrient values per portion size, we converted them to per 100 g or 100 mL. We calculated the value of any missing macronutrients by subtracting the calories obtained from the available macronutrients from the total calories and then dividing by the Atwater factor of the nutrient. For all raw foods included in our database, we added yield factors [25,26] so that the accurate nutrient values can be computed. As is common in other food composition databases such as the UK McCance and Widdowson’s dataset [26], missing micronutrient values were left blank at this stage rather than assigning a value of zero to them [6].

#### 2.1.4. Creation of Mixed Dishes

To include food composition data of mixed dishes that were not available from our data sources into myfood24, we adopted standard steps for recipe calculation within the myfood24 system [27]. We selected dishes and recipes from two local recipe books created for rural women in Nigeria [28,29]. Only the main ingredients were included to create these recipes since traditional foods in Nigeria vary greatly. The same dish can be made as a simple recipe with few ingredients or a complex version with a wider variety of ingredients due to cost, availability and ease of preparation. We compared the composition of mixed dishes in our new database with those of similar names in the UK composition tables.

#### 2.1.5. Estimation and Allocation of Portion Sizes

We allocated portion sizes to all foods using various portion estimation units. For generic raw foods, we used standard household measures such as cups and spoons. We also used commercial units for some food items, which are known by the local researchers (authors CU, HE and RS) to be commonly prepared in the same quantity as bought from the market, e.g., “1 cigarette cup of *gari*”.

In the absence of any photographic food atlas for Nigeria, one of the researchers (author CU) developed portion images using a guide [30] and experience of the Nigerian context. For soups, rice-based and legume-based dishes, the first portion was the smallest and used the smallest household measurement unit—a dessert spoon. Then, we increased the portion sizes to 4–7 by increasing the number of dessert spoons. Captured with a smartphone, images ranged from small to large to help users select the closest match to amounts consumed. Some images were used multiple times for foods with similar appearances [17]. All images were developed using standard and myfood24 photography guidelines [31].

For packaged foods, we collected portion-size units and weight per portion from the labels. Where the portion weight was not provided, we calculated it by dividing the total weight by the number of portions.

#### 2.1.6. Development of Food Accompaniments

We compiled common food pairs in Nigeria to prompt users when reporting their intake. This important feature aims to improve recall by providing suggestions for foods often eaten together. For example, sugar, honey and milk were included as food accompaniments for *ogi*; groundnut for banana, cucumber and cassava flakes; and *gari*, *fufu*, *amala* and semovita for all soups.

#### 2.1.7. Incorporation of the Database into the myfood24 System

The myfood24 team carried out quality checks throughout the database development. The database was first uploaded to a staging environment for internal testing before running them live on the system. Researchers can now access myfood24 West Africa, with a demo available on the website. To supplement the West African database, the myfood24 UK generic database, containing over 2000 foods compiled from the UK composition table [17], is also available to users.

### 2.2. Pilot Study, Feasibility and Usability Testing of myfood24

#### 2.2.1. Recruitment

This cross-sectional pilot and usability testing study of myfood24 was carried out at Michael Okpara University of Agriculture Umudike, Abia State, in southeast Nigeria. Participants, who were university staff, were recruited through flyers, oral advertisement and various WhatsApp groups of staff members. We followed established guidelines for conducting pilot studies, which recommend a minimum sample of 30 participants [32]. Eligibility criteria included an age of 18 and above, not being pregnant or having given birth to a child in the last 3 months and not being diagnosed with diabetes or taking medication to regulate blood glucose levels. Demographic characteristics were assessed using an online questionnaire. Participants were given a mobile top-up card at the end of the study as a reward for their participation.

#### 2.2.2. Dietary Assessment

Participants received a pre-recorded video tutorial to help with using myfood24. myfood24 links were sent via email and WhatsApp and we notified participants of the links through regular SMS and calls. Participants had access to the generic myfood24 UK and West Africa databases to report their dietary intake in the preceding 24 h. To maximise the time available for this study, a trained dietitian interviewed the participants who could not self-record, exploring myfood24 as an interviewer-administered tool. The feasibility of myfood24 among our participants was assessed by the total number of myfood24 food diaries submitted, whether self-recorded or interviewer-administered.

#### 2.2.3. Usability Testing

For usability testing, participants that self-recorded received a personalised online questionnaire to assess their competence in the use of technologies, previous food diary use and system usability (SUS). They rated their agreement with 10 usability statements (1 = strongly agree; 5 = strongly disagree) [33].

### 2.3. Data and Statistical Analysis

Descriptive analyses were conducted to explore the participants’ characteristics. We sorted food entries in myfood24 by database (UK or West Africa) and calculated the contribution of the West African foods to the total energy and nutrient intakes. We checked for the difference in BMI and energy intake between self-recorded and interviewed participants using the Mann–Whitney U test, and SUS score was calculated in accordance with the method proposed by Brooke [33]. The scores for each of the 10 statements ranged from 0 to 4. For odd statement numbers (1,3,5,7,9), 1 was subtracted from the scale position, while for the even statement numbers (2,4,6,8,10), 5 was subtracted from the scale position. We summed the resulting scores and multiplied by 2.5, giving an overall score ranging from 0 to 100. A score of 68 or more is regarded as “good”. Significance was set at *p* < 0.05. Analyses were performed using Stata version 17.

## 3. Results

### 3.1. myfood24 West Africa

The myfood24 West Africa version is now live on the myfood24 system and incorporates the features and functionality of the original UK version. It contains 924 commonly consumed Nigerian foods and drinks with up to 54 nutrient values per item. A list of the nutrients is provided in Table S1. Sixty percent (n = 555) of the data were sourced from the 2019 West African Food Composition Table (WAFCT), 17% (n = 155) from back-of-pack labels of packaged foods, 14% (n = 131) from the 2017 Nigeria Food Composition Table (NFCT), 5% (n = 46) from mixed dishes generated within myfood24 and 4% (n = 37) from the published literature.

As shown in Table S2, foods within the vegetable category had the highest number (n = 135, 15%) in the database followed by the meat category (n = 112, 12%); the smallest category was the fizzy drink category (n = 2, <1%). Most of the food data of items within the categories of fats and oils (22 out of 25, 88%) and milk products (32 out of 41, 78%) were sourced from back-of-pack labels.

While not in any of the FCTs used, the generated recipes and the published literature were the only data sources of food items within the soups, stews and sauces category. The ingredients used in generating the recipes were included in the food description for each of the recipes and nutrients and were computed per 100 g of the cooked food. The nutrient composition of the local dishes in our new database showed a variation of up to 200% from foods with similar names in the UK food composition database. A comparison of some of these dishes is shown in Table 1.

**Figure 2:**
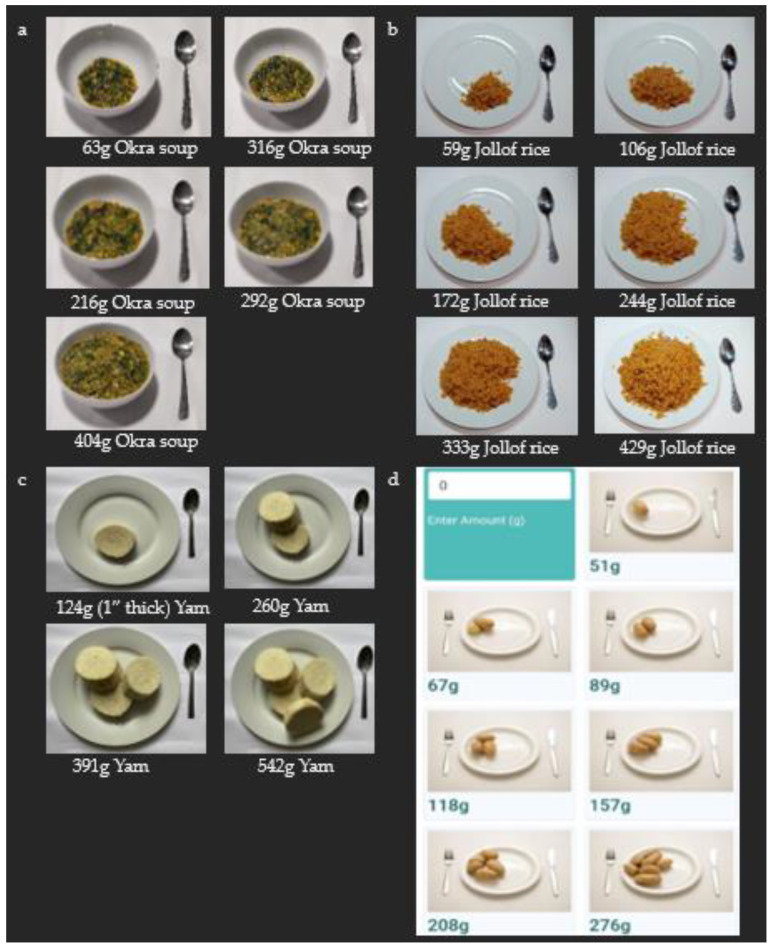
Portion images of some foods in myfood24 West Africa Database. (**a**) Okra soup; same portion images were used for all “draw soups” such as *ogbono* soup. (**b**) Jollof rice; same images were assigned to all rice-based dishes such as fried rice, concoction rice and coconut rice. (**c**) Yam, tuber, combined cultivars, boiled. (**d**) Image for new boiled potato from myfood24 UK was used for boiled cocoyam as they are similar in appearance.

Portion images were developed for 15 local foods (each with up to seven different portion sizes) in our database to help users estimate the amounts of foods they consumed. For example, some of the portion images are shown in Figure 2.

In addition, we also applied 25 foods with portion images in the original UK database to similar foods in our database. Some images were used multiple times for foods which were similar in appearance.

### 3.2. Pilot of myfood24 West Africa

With a response rate of 52%, 179 participants provided 24 h dietary recall data in the study, and the mean age was 38.5 ± 8.7 years. The majority of our participants were married (68%), had a university degree (85%) and were within the senior staff cadre (58%). The characteristics of the participants have been detailed in Table 2.

A total of 1345 food/drink entries were made with a median number of seven (IQR: 5, 9) food/drink entries per day per participant. Of the foods available for selection, gari was the most selected (n = 96, 11%), followed by tomato stew (n = 72, 8%), from our new database (Table S3).

Foods from our database contributed 60–82% of the energy and nutrient intake in our pilot study. Table 3 details the contribution of the foods in our database in the pilot study.

A total of 30% (n = 53) self-recorded their dietary intake. There was no significant difference in the energy intake (*p* = 0.67) and body mass index (*p* = 0.82) of participants who self-recorded their intake and those whose intakes were interviewed.

With a response rate of 87%, 39 participants who used myfood24 on their own completed the user questionnaire. Their mean age was 39.2 ± 8.0 years, and the majority had attended a university (92%) and had good confidence in using technology (100%) but had never self-completed or reported their food intake in the past (67%). Detailed characteristics of the participants who self-recorded their food intake are shown in Table 2. The mean system usability score (SUS) of myfood24 among our participants was 74 out of 100, indicating that it is a usable dietary assessment tool.

Some participants (n = 37) who used myfood24 on their own provided feedback on their favourite and least favourite aspects of the tool. Some emphasised the ease of use (n = 11), with one describing it as ‘very simple and easy to use’ and another praising ‘The fact that I could participate in the research from the comfort of my home’. The tool’s innovation was acknowledged by participants (n = 4), with one commending ‘The clear separation of foods eaten and drinks taken’ and another mentioning ‘It is more efficient than manual recording’. Participants (n = 5) also commended the presence of local foods, with one noting, ‘The availability of certain foods like utazi in the App’. Participants lauded the ability to estimate amounts of foods eaten using portion images included in the system (n = 7), with one commenting ‘The picture helped me select the food I ate’. The option to view a summary of total energy and macronutrients was appreciated by participants (n = 7), as one shared ‘My favourite thing about myfood24 is the nutrient summary that shows after submission of the food diary’.

On the other hand, participants noted areas of improvement. Two mentioned the absence of specific foods, with one stating ‘I didn’t find my kuli-kuli’. Another insight from five participants indicated that they would have preferred using images for portion-size estimation instead of portion weights used for some foods: ‘To input quantities of foods consumed was a bit difficult without images’. The set-up for this project using myfood24 required an internet connection, and participants were required to recall meal times. Two participants found this challenging: one stated ‘It consumes my data’ and another mentioned they struggled ‘Trying to remember the time I ate’.

## 4. Discussion

myfood24 West Africa is the first online dietary assessment tool tailored to Nigerian foods, with usability evaluated. With 954 foods, our new database contains three times as many foods than the Nigeria Food Composition Table, including traditional foods, cooked and mixed dishes and packaged foods. To reduce the high costs of food composition database development using analytical methods, we used indirect methods with data from existing sources and standardised methods. This approach, also adopted by other compilers, is valid for building food composition databases [8,34].

Our data come primarily from the 2019 WAFCT, hence the name myfood24 West Africa. While the 2019 WAFCT addressed the need for a regional food composition database [21], our work goes further to provide digital dietary assessment. We suggest collaboration between food data compilers in West Africa and the myfood24 team for updates to ensure regional representation.

Our database benefits from including branded foods, which are absent in the FCTs we used; this reflects the dietary shift from traditional to processed foods [35]. Food labels in Nigeria, regulated by the National Agency for Drug Administration and Control (NAFDAC), provide insights into improving public health [36], and incorporating the packaged foods in our database increased the variety of foods available. Regular updates are needed to capture product changes [37]. Additionally, our work provides a broader range of mixed dishes and nutrients than 2017 NFCT, which is relevant for understanding holistic dietary impact on health. Owing to socioeconomic status and religious beliefs, Nigeria’s diverse culture influences dietary habits, highlighting the need for country-specific food composition data. While some nutrient data came from the literature and 2017 NFCT, most of the data were calculated on the myfood24 system, giving a practical alternative to laboratory analysis [38,39].

Acknowledging that portion-size assessment is difficult, we have developed different portion-size options for our database. Communal eating is common in Nigeria, making portion estimation even more challenging [40]. Nigerian diets often include mixed dishes and support for portion-size estimation is required, particularly if weights are not used, to minimise measurement error. Although not all foods have portion images due to time and resource constraints, the database can be updated to include more and can possibly validate them among the population.

This study demonstrates the feasibility of using myfood24 West Africa for dietary assessment research in Nigeria, contributing at least 2/3 of the total energy and nutrient intake. No significant differences were found in the total energy intake or BMI between self-recorded and interviewer-collected data. In addition, the increasing migration of Nigerians to the West, who are less likely to change their diets [41], underscores the need for our new tool in research focused on the diets and targeted interventions of ethnic minority groups.

One-quarter of self-recording participants were nutrition professionals with experience in collecting dietary data, influencing their confidence in using the system. Lack of motivation and confidence with digital devices can reduce compliance with digital dietary records [1]. We could not assess confidence for all participants, as this information was not available for the interviewed participants. Our study highlights areas for improving myfood24 West Africa’s usability in future research. Despite a good and comparable usability score [18,19], only a third of the university participants self-recorded, and the reasons could not be investigated. Although myfood24 is quick to use [17,19], we suspect that participants perceived it as time-consuming and did not use it, likely due to staff workload post-strike. The offline feature of myfood24, which could have helped overcome internet access challenges, was not included, possibly leading to fewer people using the tool. Low response rates were also reported in a Kenyan study [42].

Our study had some strengths and limitations. We introduced myfood24 West Africa as the first online dietary assessment tool with a wider range of foods eaten in Nigeria compared to the current 2017 Nigerian Food Composition Table. Including portion sizes was crucial to help participants accurately estimate their intake—a feature lacking in other nutrition analysis software used in Nigeria. Furthermore, feedback from the myfood24 users in Nigeria gave insights for improvement. Although we could not conduct qualitative research, including quotes from the participants’ feedback gave us a better understanding on their experience with the tool [43].

However, it was not possible to conduct laboratory-based food analysis for our database and we were limited by the data available in the food composition tables, back-of-pack information as provided by food manufacturers and recipes used. Although our data on branded foods are from one region, we do not expect notable differences in these foods across the regions. Moreover, we incorporated data from food consumption surveys and the 2017 Nigerian Food Composition Table, which capture geographical variations. While our database lacks information on branded alcoholic drinks and foods sold in fast-food outlets in Nigeria, some of these fast-food items are similar to local or Western dishes already included in our new database and the UK database, respectively. Generic data on alcohol is available on the myfood24 UK database. Nevertheless, myfood24 allows for database updates as more information becomes available. The portion-size images of the mixed dishes in our new database were not validated, which may mean they do not accurately reflect the actual quantities of the foods selected in the pilot study. In addition to providing at least four images to the participants, strict guidelines for weighing and photographing portion images were followed to allow participants to accurately estimate the amounts consumed. Nevertheless, myfood24 allows for database updates as more information becomes available. Future research will include updates with more foods within the country and region as well as the validation of the portion sizes. Whilst we aimed to capture individuals of different educational backgrounds within our sample, we were limited by the number of participants in our study. This study was conducted at a difficult political time in Nigeria and university staff were returning from an overstretched period of industrial action. Although these factors may have contributed to the insufficient robustness of our samples, the use of myfood24 as an interviewer-administered tool for the majority of our participants helped mitigate the impact of this limitation on the use of the system. In addition, while this was a pilot feasibility study and not conclusive, it serves as a step towards more research in the future. However, our study is important considering there is extremely limited information on the dietary intake in Nigeria using detailed measurement tools. Although we did not consider this for our study, we recommend using the offline version of myfood24 in future research in Nigeria and West Africa to increase participants’ accessibility.

## 5. Conclusions

This paper described how we developed myfood24 West Africa, a technology-based dietary assessment tool that is now live on the myfood24 system. The database comprises 924 culturally relevant foods in Nigeria and was compiled using various sources, including the West Africa Food Composition Table, the Nigeria Food Composition Table, back-of-pack labels of branded foods, published articles on nutritional composition of foods and recipe calculations using the myfood24 system. With the improvements suggested in this study, the validation of portion sizes and the addition of other West African foods in further updates, myfood24 will be a valuable research tool for investigating the relationships between diet and health outcomes among West Africans, both within West Africa and in the diaspora.

## Figures and Tables

**Table 1:** Nutrient composition of selected mixed dishes in the myfood24 West Africa versus UK databases.

Components	Food Name													
	Beef Pastry ^1^		Vegetable Soup ^2^		Okra Dish ^3^		Fried Rice ^4^		Rice and Beans Dish ^5^		Rice Dish ^6^		Tomato Sauce ^7^	
	WA	UK	WA	UK	WA	UK	WA	UK	WA	UK	WA	UK	WA	UK
Energy (kcal)	213	292	101	55	34	98	173	169	137	170	137	147	196	89
Protein (g)	5.1	9.2	5.0	1.0	1.7	3.0	2.6	3.9	3.0	5.6	2.0	2.9	1.0	2.2
Fat (g)	11.3	17.7	6.8	4.2	2.7	7.7	3.4	5.3	7.0	2.2	6.2	2.6	18.9	5.5
Carbohydrates(g)	24.4	25.5	3.8	3.7	0.7	4.7	32.6	28.1	17.0	34.1	19.1	27.9	5.2	8.6
Fibre (g)	1.3	2.1	1.9	N	0.6	N	1.1	2.8	0.3	N	0.6	0.7	1.6	-
Sodium (mg)	308.6	332.0	357.6	315.0	176.3	25.0	5.7	409.0	0.0	15.0	348.0	326.0	498.0	340.0
Calcium (mg)	44.0	41.0	229.8	14.0	87.0	158.0	3.5	28.0	0.0	19.0	16.5	8.0	15.0	19.0
Iron (mg)	0.9	1.1	6.3	0.3	0.7	1.4	1.4	0.4	0.8	1.3	0.1	0.7	0.3	0.6
Vitamin A (µg) *	104.3	N	143.9	245.0	10.6	109.0	22.0	4.0	0.0	N	16.3	9.0	86.1	204.0
Folate (µg)	8.6	2.0	11.5	6.0	13.2	45.0	6.0	8.0	0.0	50.0	9.8	6.0	15.7	9.0
Vitamin C (mg)	0.4	N	86.6	2.0	3.9	13.0	0.0	tr	0.0	tr	4.1	3.0	12.4	8.0
% difference	7–125%		3–182%		55–165%		2–194%		0–104%		7–150%		24–110%	

* retinol equivalent; WA—myfood24 West Africa; UK—UK composition of foods integrated dataset; tr—trace value; N—reliable information on the amount is lacking; 1—for WA: meat pie (beef); for UK: pie, beef, puff or shortcrust pastry, individual, retail; 2—for WA: vegetable soup; for UK: soup, vegetable, homemade; 3—for WA: okra soup; for UK: okra with tomatoes and onion, West Indian, homemade; 4—for WA: fried rice; for UK: rice, egg fried, ready cooked, re-heated, retail, not takeaway; 5—for WA: jollof rice and beans; for UK: rice and black-eyed beans; 6—for WA: jollof rice; for UK: pilaf, rice with tomato, homemade; 7—for WA: tomato stew; for UK: tomato sauce, homemade.

**Table 2:** Characteristics of the pilot study participants.

Characteristics	All Participants (n = 179)	Self-Administered myfood24, n = 53 (30%)	Interviewer-Administered myfood24, n = 126 (70%)
Age, years (mean (SD))	41.2 (9.2)	38.5 (8.7)	42.7 (9.2)
	n (%)	n (%)	n (%)
Age, years			
20–29	15 (8)	8 (15)	7 (6)
30–39	62 (35)	21 (40)	41 (33)
40–49	69 (39)	20 (38)	49 (39)
50–59	27 (15)	3 (6)	24 (19)
60–69	6 (3)	1 (1)	5 (4)
Gender			
Male	85 (47)	25 (47)	60 (48)
Female	95 (53)	28 (53)	66 (52)
Marital status			
Currently single	57 (32)	20 (38)	37 (29)
Married	122 (68)	33 (62)	89 (71)
Place of residence			
Rural	63 (35)	15 (23)	48 (38)
Urban	116 (65)	38 (33)	78 (62)
Job rank			
Junior non-teaching	28 (16)	6 (11)	22 (17)
Senior non-teaching	104 (58)	23 (44)	81 (65)
Teaching	47 (26)	24 (45)	23 (18)
Educational level			
Secondary or less	15 (8)	3 (6)	12 (10)
Post-secondary	13 (7)	2 (4)	11 (9)
Graduate/Postgraduate	151 (85)	48 (90)	103 (81)
Profession			
Non-nutritionists (%)	167 (93)	41 (77)	126 (100)
Nutritionists (%)	12 (7)	12 (23)	0 (0)
Religion			
Christianity	177 (98)	53 (100)	124 (98)
Islam	1 (1)	0 (0)	1 (1)
Others	1 (1)	0 (0)	1 (1)
Smoking status			
Current smoker	1 (1)	0 (0)	1 (1)
Non-smoker	178 (99)	53 (100)	125 (99)
Alcohol intake			
Current drinker	113 (63)	37 (70)	76 (60)
Non-drinker	66 (37)	16 (30)	50 (40)
Body mass index			
Underweight	5 (3)	1 (2)	4 (3)
Normal	59 (33)	17 (32)	42 (33)
Overweight	62 (35)	19 (36)	43 (34)
Obese	53 (29)	16 (30)	37 (30)

**Table 3:** Percentage contribution by food type of myfood24 West Africa database to energy and nutrient intakes.

Nutrients	Food Groups																
	Bread (5/38)	Snacks (34/116)	Cereals (106/64)	Spices (37/13)	Fats (25/40)	Fish (61/83)	Fizzy Drinks (2/7)	Fruits (44/95)	Pulses (89/44)	Meat (112/227)	Milk (41/132)	Nuts (45/25)	Soups (35/58)	Roots/ Tubers (95/32)	Sugar (20/72)	Veg (135/125)	Total (95% CI)
Energy	0	4	14	0	1	1	1	1	6	1	2	3	12	30	1	0	78 (66,80)
Protein	0	3	13	0	0	5	0	1	11	6	3	4	15	11	1	0	73 (66,80)
Fat	0	5	12	0	3	2	0	1	8	2	3	5	32	8	1	0	82 (78,87)
Carbohydrate	0	4	16	0	0	0	2	2	5	0	1	1	3	40	2	0	76 (69,82)
Fibre	0	4	9	0	0	0	0	2	6	0	0	2	18	26	1	1	70 (63,76)
Sodium	0	5	6	0	0	2	1	0	3	1	1	0	42	13	0	0	74 (67,80)
Iron	0	2	14	0	0	2	0	1	7	4	1	1	16	29	3	1	81 (74,86)
Vitamin A	0	8	4	0	5	2	0	3	2	3	8	0	30	4	1	1	71 (64,77)
Folate	2	4	7	0	0	2	3	1	5	1	3	1	20	12	1	0	60 (52,67)
Vitamin C	0	1	3	0	0	0	0	2	3	2	2	0	34	17	4	2	70 (63,76)
Number of foods items *	2	44	115	7	11	39	21	27	64	76	33	38	193	185	37	17	909

Veg, vegetables. The figures beneath the food groups represent the number of foods in our new West Africa and UK generic databases. For example, there are 5 and 38 bread products in our new WA and UK generic databases, respectively. Snacks include pastries, cakes and biscuits. Sugar includes confectioneries and powdered sweetened drinks. Eggs and alcoholic drinks were not selected from the WA database. * Number of food items consumed and selected from the WA database by food type by participants.

## Data Availability

Data are contained within the article and Supplementary Materials.

## Supplementary Materials

The following supporting information can be downloaded at: https://www.mdpi.com/article/10.3390/nu16203497/s1, Table S1: List of nutrients available in the myfood24 West Africa; Table S2: Food categories in the database by sources; Table S3: Top 10 foods selected from the myfood24 West Africa database.
