# Supplementary material for: Development and Pilot Study of myfood24 West Africa—An Online Tool for Dietary Assessment in Nigeria
Source: Nutrients. 2024 Oct 15;16(20):3497. doi: 10.3390/nu16203497 (PMC11509910; doi:10.3390/nu16203497)
Supplement: Supplementary file 1 [file nutrients-16-03497-s001.zip › nutrients-3226061-supplementary.pdf]

Supplementary Materials

Table S1. List of nutrients available in the myfood24 West Africa.

Nutrients included in the myfood24 West Africa database	
Energy (kcal)	Chloride (mg)
Energy (kj)	Manganese (mg)
Water	Selenium (mg)
Total nitrogen (g)	Retinol (μg)
Protein (g)	Beta-carotene (μg)
Fat (g)	Retinol Equivalent (μg)
Carbohydrates (g)	Vitamin D (μg)
Starch (g)	Vitamin E (μg)
Total sugar (g)	Vitamin K1 (μg)
Glucose (g)	Thiamin (mg)
Fructose (g)	Riboflavin (mg)
Sucrose (g)	Niacin (mg)
Maltose (g)	Trytophan/60 (mg)
AOAC fibre (g)	Niacin Equivalent
Saturated fatty acid per 100g of food (g)	Vitamin B6 (mg)
Monosaturated fatty acid per 100g of food (g)	Vitamin B12 (mg)
Polysaturated fatty acid per 100g of food (g)	Folate (μg)
Total trans fatty acid per 100g of food (g)	Pantothenate (μg)
Cholesterol (mg)	Biotin (mg)
Sodium (mg)	Vitamin C (mg)
Potassuim (mg)	Alpha-carotene (μg)
Calcium (mg)	Beta-carotene (μg)
Magnesium (mg)	Cryptoxanthins (μg)
Phosphorus (mg)	Alpha-tocopherol (μg)
Iron (mg)	Beta-tocopherol (μg)
Copper (mg)	Delta-tocopherol (μg)
Zinc (mg)	Gamma-tocopherol (μg)

Table S2. Food categories in the database by sources.

	Data sources					
**Food category**	BOP labels (n 155)	NFCT (n 131)	WAFCT (n 555)	Literature (n 37)	Recipes (n 46)	Total (n 924)
Alcoholic drinks	0	5	7	0	0	12
Bread	0	0	5	0	0	5
Cakes, biscuits, pastries & other snacks	25	0	2	0	6	33
Cereals & products	24	6	67	4	5	106
Condiments & spices	6	7	22	2	0	37
Eggs & products	0	2	13	0	0	15
Fats & oils	22	0	3	0	0	25
Fish & products	0	7	54	0	0	61
Fizzy drinks	0	0	1	1	0	2

Fruits & products	9	15	19	1	0	44
Legumes & products	1	12	72	0	4	89
Meat, poultry & products	1	27	84	0	0	112
Milk & products	32	7	2	0	0	41
Nuts, seeds & products	7	10	24	3	1	45
Other drinks	3	2	7	0	0	12
Soups, stews & sauces	0	0	0	12	23	35
Starchy roots, tubers & products	8	9	63	8	7	95
Sugar, sweets & chocolate drinks	12	2	6	0	0	20
Vegetables & products	5	20	104	6	0	135
**Total**	**155**	**131**	**555**	**37**	**46**	**924**

WAFCT, West Africa Food Composition Table; NFCT, Nigerian Food Composition Table; BOP, Back-of-pack labels of packaged foods

Table S3. Top 10 foods selected from the myfood24 West Africa database.

S/N	Food name	N (%)
1	Cassava, grated, from fermented white cassava, toasted without oil (white gari)	96 (11)
2	Tomato stew, cooked with tomato concentrate, fresh tomatoes, onion, vegetable oil and salt, without meat and fish	72 (8)
3	Groundnut, roasted	31 (3)
4	Rice cooked with fresh tomatoes, tomato concentrate, onion, vegetable oil, salt, without meat or fish	30 (3)
5	Melon seed soup, with beef and stockfish, vegetables, no thickener	24 (3)
6	Beans, whole, boiled with oil	22 (2)
7	Rice, white, boiled (without salt), drained	22 (2)
8	Beef meat, lean, ca. 5% fat, raw	21 (2)
9	Buns, fried	19 (2)
10	Mackerel, fried, with bones	19 (2)
